# Editorial for the Special Issue “Application and Modification of Clay Minerals”

**DOI:** 10.3390/ma18245527

**Published:** 2025-12-09

**Authors:** Gang Yang

**Affiliations:** College of Resources and Environment, Southwest University, Chongqing 400715, China; theobiochem@163.com

Clay minerals, a diverse group of hydrous aluminosilicates, are ubiquitously distributed and primarily comprise soils, sediments, and sedimentary rocks. The nanoscale layered architecture endows them with distinctive properties such as high specific surface area, superior cation exchange capacity, and unique plastic, rheological, and swelling behaviors. These underpin their widespread applications, with recent advances being particularly noteworthy. Through modification and targeted synthesis, clay-based materials can be tailored with special attributes to meet specific functional demands, and have transcended their traditional applications in ceramics and construction and entered advanced, cutting-edge fields [1,2]:

1. **Environmental Remediation and Sustainable Materials:** Clay minerals function as low-cost and efficient scavengers for a wide spectrum of contaminants, including heavy metals, volatile organic compounds, dyes, pesticides, and pharmaceuticals. In addition to the elimination of existing contaminants, one of the core objectives is to prevent future environmental damage through the construction of “green” materials and processes. This aligns perfectly with the design of sustainable materials with minimized environmental footprint.
2. **Catalysis:** Clay minerals are utilized directly as acid catalysts or after activation by mineral acids like H_2_SO_4_. Pillared interlayered clays (PILCs) constitute the cornerstone of advanced clay catalysis, manufactured via the intercalation of large polyoxycations into clay interlayers and then calcination. PILCs possess stable, zeolite-like pore architecture, with high surface area and strong acidity, and serve as desired catalysts for petroleum cracking, exhaust purification, and other processes. Additionally, clay minerals serve as good supports for metals or metal oxide nanocatalysts.
3. **Advanced Materials and Nanocomposites:** Clay minerals, as high-aspect-ratio platelets with tremendous surface area and modifiable surface chemistry, are regarded to be pivotal components of nanoscale materials and composite. Through strategic ion exchange or organo-modification, clay minerals can be rendered compatible with polymer matrices, and the resulting composites exhibit multifaceted enhancements, such as reinforced mechanical strength and modulus, superior thermal stability and flame retardancy, and reduced gas permeability.

This Special Issue, “Application and Modification of Clay Minerals,” published in *Materials*, was conceived to address these challenges and showcase the cutting-edge research pushing the boundaries across diverse fields. The collected contributions aim to present a compelling portfolio of research that advances our knowledge on advanced synthesis routes, innovative modification, and potential applications in environmental remediation, materials science, and industrial processes. In what follows, I will briefly review all fourteen papers published in this Special Issue according to the aforementioned classification approach.

1. **Environmental Remediation and Sustainable Materials:** Nartowska et al. examine the impacts of ZnCl_2_ contamination on the property and structural alterations of Na- and Ca-bentonites, which impairs the performance of bentonites as sealing barriers. Exposure of bentonites to concentrated ZnCl_2_ solutions triggers the formation of a new mineral phase called simonkolleite (~30%). The extensive ion exchange between Zn^2+^ and Na^+^/Ca^2+^ markedly reduces the Si, Al, Mg, and Fe contents within bentonite, and the uniform distribution of Zn and Cl across the entire bentonite surface induces the formation of the simonkolleite phase, which, alongside the structural changes within bentonite, adversely affects bentonite’s function, such as through reduction in surface area and porosity, and compromises its performance as a sealing barrier. Zhao et al. focus on the development of layered double hydroxides (LDHs)-based materials for wastewater treatment. The active surface, which often becomes deactivated during standard preparation and drying processes, is preserved intact in the synthesized NiFe-LDH adsorbent by being kept in a wet state. NiFe-LDH is highly efficient for the removal of methyl orange from wastewater, and the adsorption capacity reaches as high as 506.30 mg/g. This is attributed to a dual mechanism of surface adsorption and catalytic degradation, and approximately half is subject to degradation. Surface hydroxyls and vacancies are the key functional elements; hydroxyls promote adsorption, while vacancies, as electron-rich centers, are crucial to generating the degrading radicals (O_2_^•−^).

To integrate sustainability into construction materials, Fang et al. replace the scarce freshwater and river sand with seawater and sea sand to manufacture concrete. However, the abundant Cl^−^ ions pose a dual challenge, which can refine the concrete’s microstructure through the shape of Friedel’s salt phases, which also leads to the formation of damaging delayed ettringite. Increasing the Cl^−^ concentrations enhances both Friedel’s salt and delayed ettringite, while the elevation of curing temperatures or the addition of nano-metakaolin promotes Friedel’s salt and suppresses delayed ettringite, thus effectively mitigating the adverse effects of Cl^−^. In another study, the authors from the same group demonstrate the effects of Cl^−^ concentration, fly ash, and curing temperature on the microstructural evolution of two primary cement hydration products: C-S-H gels and calcium hydroxide. The presence of Cl^−^ ions increases the concentration of Ca^2+^ compounds and favors the production of C-S-H gels. Fly ash reacts with calcium hydroxide generated via cement hydration to produce C-S-H gels, and 50 °C curing can stimulate the activity of fly ash, generating a large amount of C-S-H gels. The contents of C-S-H gel and calcium hydroxide have the largest gray correlation with fly ash dosage. Among these factors, fly ash achieves the highest correlation with the amount of C-S-H gels. Maosa et al. reveal that mechanical activation is effective for preparing clay minerals to be used as supplementary cementitious materials for the formulation of low-carbon cements. Using the response surface methodology, the various grinding parameters are optimized to achieve a high degree of clay activation. The high rotation speed and prolonged grinding time promote pozzolanic activity by boosting the formation of amorphous phases from clay minerals and reducing the particle size. However, the intermediate milling parameters are sufficient to achieve the substantial degrees of amorphization and pozzolanic activity that further grant the activated clay’s reactivity. The exceedingly aggressive milling introduces impurities from the milling equipment wear, and a balance is essential.

2. **Catalysis:** Yu et al. manufacture a series of iron-rich red mud catalysts under a wide range of calcination temperatures (300~1100 °C). The catalysts exhibit superior activity for the combustion of biodiesel wastewater when calcinated below 400 °C. A temperature of 350 °C is optimal, and the removal rate of chemical oxygen demand reaches 100%. Calcination increases the specific surface area of catalysts to supply more active sites and develop larger pore structures that further promote adsorption and diffusion. Dispersion of α-Fe_2_O_3_ at 350 °C is higher than in other calcination temperature catalysts, and a large quantity of Fe^3+^ and metal–oxygen is distributed on the surface of the catalysts, which significantly lowers the reduction temperature and enhances catalytic reactivity. This study provides insightful clues for the preparation of clay-based catalysts and offer a sustainable solution for waste valorization.
3. **Advanced Materials and Nanocomposites:** Lahchich et al. establish the relationships between the physicochemical and structural properties of five commercial vermiculites from different sources, which are crucial for industrial applications, particularly for thermal exfoliation. The mechanochemical treatment significantly enlarges specific surface area and pore volume, while higher crystallinity reduces specific surface area but improves mechanical strength and thermal stability. The chemical composition of vermiculites influences thermal expansion, while temperature and intensity of the exfoliation process rely on such factors as particle size and the identification of interlayer cations. Green schist is a typical heterogeneous material composed primarily of quartz, chlorite, and muscovite, and Wang et al. conduct systematic femtosecond laser scanning experiments on green schist to fabricate microgrooves under various conditions. The green schist of a transverse rather than a longitudinal section has a more uniform composition and is suitable for micro/nanostructure fabrication. The optimal processing parameters (60 μm groove spacing and 2~6 scan passes) achieve stable superhydrophobic performance.

Koroleva et al. address the impacts of different silica precursors, pH conditions, and synthesis durations on the synthesis of kaolinite group minerals, and pH exerts the most significant alteration, with the formation of halloysite with a small admixture of kaolinite at a high pH and the formation of ordered kaolinite at a low pH. Yang et al. propose a new micronization approach to disaggregate the bundles of sepiolite while preserving the original structural integrity of fibers. The sepiolite powders with a mass ratio of more than 90% and particle size of less than 22 μm are readily obtained at a steam pressure of 0.6 MPa. The steam pressure change method shows obvious advantages over mechanical extrusion in maintaining fiber integrity and activating the surface, thus showing potential applications as scavengers. Wang et al. manufacture the low-silicon X-type zeolite from lithium slag without extra Si and Al sources. The X-type zeolite has a high ion exchange capacity for Mg^2+^ of 191 mg MgCO_3_/g, two times that of 4A zeolite, and a high ion exchange capacity for Ca^2+^ of 302 mg CaCO_3_/g, which meets the first-grade standard of zeolite for detergent builders. With sea sand as the Si source and Al(NO_3_)_3_ as the Al source, Xie et al. manufacture analcime with the tunable Si/Al ratios. Impurities such as quartz and sodalite are detected when using acid-treated sea sand, while pure analcime is readily available when using alkali-treated sea sand. The adsorption capacity of Cu^2+^ amounts to 64.8 mg/g, and the adsorption kinetics follow the pseudo-second-order model.

A meso–macroporous analcime/sodalite zeolite composite is produced by Esaifan et al. by means of a hybrid synthesis process employing both a complex template method and the hydrothermal treatment of naturally abundant kaolinitic-rich clay. There are two types of zeolite particles: a hollow microsphere with an analcime icositetrahedron of 30~40 µm and a sodalite microsphere with a ball-like morphology of 3~4 µm. The composite exhibits excellent scavenging capacities for a wide range of pollutants (inorganic Cu, Cd, Cr, Ni, Zn, and Pb ions, organic dyes, phenolic compounds, and surfactants) and superior catalytic degradation activity for volatile organic compounds. Liang et al. conduct the intercalation of sericite with anionic surfactants rather than with the widespread cationic surfactants, following thermal modification, acid activation, and sodium modification procedures. Sericite, with the co-intercalation of cationic surfactant hexadecyl trimethyl ammonium bromide (CTAB) and anionic surfactant sodium dodecyl sulfate (SDS), has a layer-to-layer distance of 6.56 nm and an intercalation rate of 95.7%. Subsequently, the sericite/epoxy composite is prepared, and it exhibits enhanced mechanical properties when compared to epoxy resin, showing potential applications in oil well drilling and paints.

The definitive classification of these papers is difficult given that many encompass interdisciplinary fields. The paper of Esaifan et al. exemplifies such an overlap, and although attributed to “Advanced Materials and Nanocomposites,” it is also highly relevant to “Environmental Remediation and Sustainable Materials” and “Catalysis.” The pursuit of interdisciplinary research can lead to the discovery of new investigative avenues. Looking forward, the following research directions should be prioritized to advance the field of clay mineral science and engineering [3,4,5]:

1. **Green Catalysis:** Benefiting from natural abundance and an eco-friendly profile, clay minerals are desirable for green catalysis and function as potent catalysts or supports in biodiesel production and organic pollutants degradation. The future of clay-based catalysts lies in smart design, leveraging them as the pivotal materials for developing sustainable and economically feasible chemical processes.
2. **Advanced Materials and Nanocomposites:** Clay minerals have secured a place in the next generation of advanced materials and nanocomposites. Through precise surface engineering, clay minerals are readily transformed into active components for separation membranes, smart coatings, flexible electronics, and other purposes.
3. **Energy Storage and Conversion:** In energy storage, clay minerals are engineered as key components of the next generation of batteries and supercapacitors and serve as templates or hosts for electrode materials. In energy conversion, clay-based materials function as efficient catalysts or supports in biomass conversion and water splitting for H_2_ production and other catalytic processes.
4. **Biomedical and Healthcare:** Clay minerals have emerged as versatile and biocompatible materials and are widely employed as engineered nanocarriers for controlled drug delivery. In addition, their inherent antibacterial properties are harnessed for topical wound healing and combating infections.
5. **Tissue Engineering Scaffolds:** Clay minerals are revolutionizing tissue engineering as multifunctional reinforcing agents in scaffold design. Their incorporation into biopolymer hydrogels notably enhances their mechanical properties and promotes cell adhesion for bone and skin regeneration.
6. **Agriculture and Food Security:** Similar to drug delivery, clay minerals can be engineered to encapsulate fertilizers and pesticides and cause them to release slowly in response to soil conditions, thus enhancing nutrient-use efficiency and minimizing environmental runoff. Furthermore, clay minerals are added to animal feed to bind toxins and enhance gut health, thereby boosting livestock productivity.

Clay minerals are poised for a renaissance, and a transformation is underway marking a shift from bulk, low-tech applications to high-value, engineered nanomaterials. The future lies not simply in their inherent properties but in our growing ability to manipulate their structure at the nanoscale. The interdisciplinary synergy of materials science, nanotechnology, chemistry, and biotechnology with clay research is poised to unlock unprecedented opportunities, thereby elevating clay minerals to the status of foundational elements in forging a sustainable and healthy society.

Finally, I would like to extend my sincere gratitude to all the authors, reviewers, and the editorial team of *Materials* whose hard work and dedication made this Special Issue possible.
